# Efficacy and Safety of Rituximab in Antiglomerular Basement Membrane Disease

**DOI:** 10.1016/j.ekir.2024.12.026

**Published:** 2024-12-31

**Authors:** Vanja Ivković, Ingeborg Bajema, Annette Bruchfeld, Stephen McAdoo, Asheesh Kumar, Richard Klaus, Nele Kanzelmeyer, Maxime Touzot, Georgina Maalouf, Ajay Jaryal, Sanjay Vikrant, Dieter Haffner, Bärbel Lange-Sperandio, David Saadoun, Mårten Segelmark, Andreas Kronbichler

**Affiliations:** 1 Department of Health, Medicine and Caring Sciences, Linköping University, Linköping, Sweden; 2 Faculty of Health Studies, University of Rijeka, Rijeka, Croatia; 3 Department of Pathology and Medical Biology, University Medical Center, University of Groningen, Groningen, Netherlands; 4 Department of Renal Medicine, Karolinska Institutet CLINTEC, Karolinska University Hospital, Stockholm, Sweden; 5 Centre for Inflammatory Disease, Department of Immunology and Inflammation, Imperial College London, Hammersmith Campus, London, UK; 6 Imperial College Renal and Transplant Centre, Imperial College Healthcare NHS Trust, Hammersmith Hospital, London, UK; 7 Department of Nephrology, All India Institute of Medical Sciences, Vijaypur, Jammu, India; 8 Department of Pediatrics, Dr. von Hauner Children's Hospital, LMU University Hospital, LMU Munich, Munich, Germany; 9 Department of Pediatric Kidney, Liver and Metabolic Diseases, Hannover Medical School, Hannover, Germany; 10 Center for Rare Diseases, Hannover Medical School, Hannover, Germany; 11 AURA Paris Plaisance, dialyse et aphérèse thérapeutique, Paris, France; 12 Department of Internal Medicine and Clinical Immunology, Sorbonne Universités, Assistance Publique-Hôpitaux de Paris, Pitié-Salpêtrière University Hospital, Centre national de références Maladies Autoimmunes et systémiques rares, National Reference Centre for Rare Autoinflammatory Diseases and Inflammatory Amyloidosis, INSERM, UMR S959, Immunology-Immunopathology-Immunotherapy, Paris, France; 13 Department of Medicine, All India Institute of Medical Sciences, Bilaspur, Himachal Pradesh, India; 14 Department of Nephrology, All India Institute of Medical Sciences, Bilaspur, Himachal Pradesh, India; 15 Department of Clinical Sciences, Lund University, Lund, Sweden; 16 Department of Endocrinology, Nephrology and Rheumatology, Skåne University Hospital, Lund, Sweden; 17 Department of Internal Medicine IV, Nephrology and Hypertension, Medical University Innsbruck, Innsbruck, Austria

**Keywords:** anti–glomerular basement membrane disease, glomerulonephritis, rituximab

## Abstract

**Introduction:**

Anti–glomerular basement membrane (GBM) disease is caused by pathogenic antibodies usually targeting the noncollagenous domain of the α3 chain of type IV collagen and frequently presents as rapidly progressive glomerulonephritis and diffuse alveolar hemorrhage (DAH). Rapid reduction of these antibodies is imperative for kidney survival and the mainstay of therapy is the combination of plasma exchange (PLEX), glucocorticoids, and cyclophosphamide. Rituximab has been postulated as a potential treatment for anti-GBM disease; however, data on efficacy and safety are lacking.

**Methods:**

We performed a review of case reports and series (*n* = 28) providing individual patient-level data on the efficacy and safety of rituximab in adult and pediatric anti-GBM disease. In addition, we have received data from authors on 18 patients which stem from 4 studies that did not report on patient-level outcomes or were not previously reported. A search strategy of studies indexed in PubMed/MEDLINE was performed, followed by synthesis and analysis of the data.

**Results:**

Sixty-seven patients [37 female (55%); 14 pediatric (21%); median age: 37 years] were followed-up with for a total of 87.1 person-years (median follow-up time: 9.5 months). They received rituximab as first-line (*n* = 39) or second-line (*n* = 28). Median serum creatinine was 416 μmol/l with 32 patients (48%) being dialysis-dependent at presentation and 24 (36%) having DAH. Intravenous pulse, oral glucocorticoids and PLEX were used in 85%, 98%, and 93%, respectively; and 54% of them received cyclophosphamide. Patients received a median of 4 (2–4) doses of rituximab with 11 patients (16%) having transient adverse effects. Patient survival was 91% and kidney survival was 67% (53% in adults and 71% in pediatric patients). Kidney survival was lower in initially dialysis-dependent patients (34% vs. 81%, *P* < 0.001). Patients receiving second-line rituximab had better kidney survival compared with those receiving it as first-line (73% vs. 46%, *P* = 0.03).

**Conclusion:**

Acknowledging the limitations of our study, including publication and selection bias, rituximab had a favorable toxicity and efficacy profile. The results indicate that rituximab can be considered as a second-line therapy in anti-GBM disease when cyclophosphamide is contraindicated.

## Graphical abstract

**Figure:**
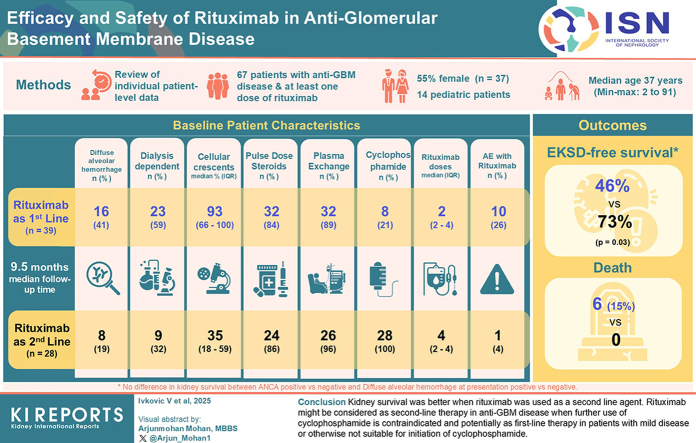

Anti-GBM disease is a rare small vessel vasculitis caused by pathogenic antibodies usually targeting the noncollagenous domain of the α3 chain of type IV collagen, a constituent of several basement membranes, most notably those of glomerular and alveolar capillaries. It frequently manifests with a clinical picture of pulmonary-renal syndrome characterized by rapidly progressive glomerulonephritis and DAH. Anti-GBM disease is typically treated with a combination of PLEX, corticosteroids, and cyclophosphamide. The disease rarely affects children, who generally have better outcomes than adults. If left untreated, the disease invariably has a dire outcome.^1^, ^2^, ^3^, ^4^, ^5^

Although treatment results in excellent overall survival rates, kidney survival varies. In one study, patients with initial serum creatinine < 500 μmol/l had 95% and 94% 1-year and 5-year kidney survival, respectively. Compared with this, those who had a serum creatinine ≥ 500 μmol/l, but were not dialysis-dependent had an 82% and 50% 1-year and 5-year survival, respectively, whereas dialysis-dependent patients had an 8% 1-year survival rate.^1^^,^^6^ The patient and kidney survival rates reported in other studies tend to be lower, which probably reflects selection of certain patient cohorts, that is, exclusion of double-positive cases, defined as presence of antineutrophil cytoplasmic antibodies (ANCA) and anti-GBM antibodies. Therapy with cyclophosphamide is associated with significant toxicity frequently causing abnormalities in the white blood cell count, severe infectious complications (especially when given in addition to glucocorticoids), and is associated with a high risk of secondary malignancy, especially when higher doses are used.^7^^,^^8^ Thus, it has become important to identify patients with anti-GBM disease who will eventually not benefit from long-term treatment with high-dose glucocorticoids and cyclophosphamide. Scenarios where avoidance of cyclophosphamide are not uncommon, which include frail patients, and where preservation of fertility is desired, are highly valued.^9^^,^^10^ Recent treatment approaches have shifted to rituximab as a potential therapeutic option in anti-GBM disease. Rituximab is a monoclonal antibody targeting CD20, which has proven to be safe and efficacious in a number of renal diseases; however, evidence in anti-GBM disease is lacking.^11^, ^12^, ^13^, ^14^ Currently, the best evidence on its use in anti-GBM disease stems from a 2018 case-based review, which included 22 cases and a recent retrospective study which compared rituximab (*n* = 14) with cyclophosphamide (*n* = 11) that had several important limitations.^15^^,^^16^ However, to the best of our knowledge, no study has systematically analyzed patient-level data from case reports and series to evaluate the efficacy and safety of rituximab in anti-GBM disease.

## Methods

**Figure 1:**
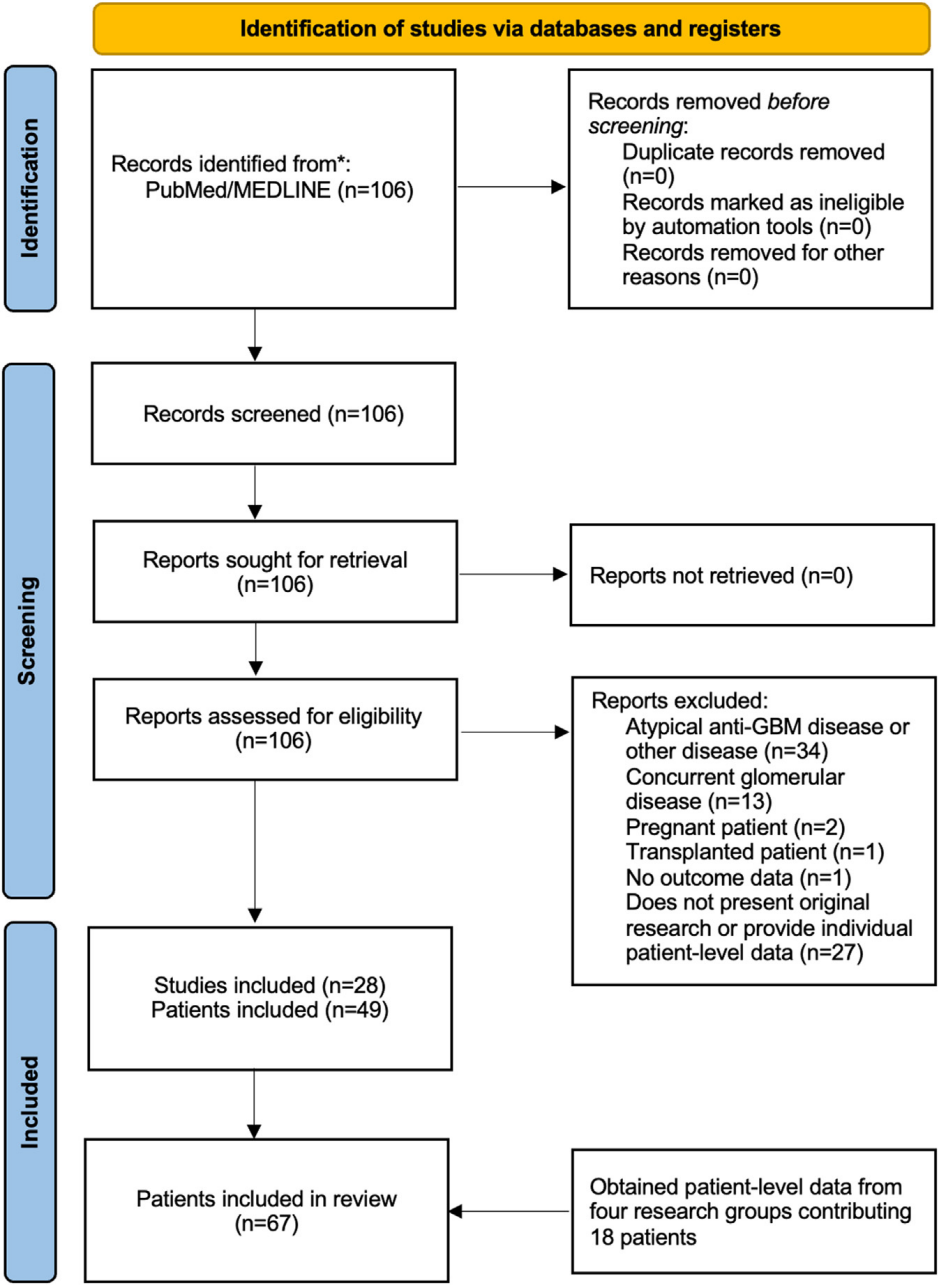
Flow diagram of included studies.

We devised a search strategy of studies indexed in PubMed/MEDLINE published up to December 10, 2024 using a search string containing the following terms: “anti-GBM disease,” “anti-glomerular basement disease,” “Goodpasture’s syndrome,” “double positive disease,” “double positivity,” and “rituximab,” which resulted in an initial pool of 106 studies. These studies were further filtered to select studies (mainly case reports and series) reporting individual patient-level data on patients with anti-GBM disease and receiving rituximab as first-line of treatment. This was defined as patients who did not receive cyclophosphamide previously, for this current episode of disease, whereas concomitant use of cyclophosphamide and rituximab was allowed; or second-line therapy, defined as patients who have received at least 1 dose of cyclophosphamide before rituximab in this episode of disease (Figure 1, Supplementary list of included studies). A total of 28 studies reporting on 49 patients were included. We included double-positive patients (concurrent presence of ANCA-associated vasculitis and anti-GBM disease), and excluded cases of pregnant women, kidney allograft recipients, patients with primary kidney disease other than anti-GBM disease, patients with seronegative anti-GBM disease, patients with concurrent other glomerular disease, as well as those without any data on the outcome. After this, we contacted the corresponding authors of studies which fit our inclusion criteria, but did not provide patient-level data. All but 1 responded and have contributed with patient-level data on 18 more patients for a total of 67 patients (53 adult and 14 pediatric cases; 39 receiving rituximab as first-line and 28 as second-line).

In general, the definitions of severe and refractory disease were not standardized or uniformly reported among studies. Most studies defined severe disease as dialysis-dependence at diagnosis, the percentage of crescents ≥ 90%, or the need for mechanical ventilation because of DAH. Refractory disease was defined as no response to standard therapy showcased by persistent antibody positivity, relapse (immunological and clinical) or complications of therapy which warranted change in treatment.

Continuous variables were tested for normality of distribution using Kolmogorov-Smirnov test and presented, depending on this, as mean and SD and median and interquartile range (IQR), whereas categorical data were presented as absolute value and percentage. Student *t-*test for unpaired samples and Mann-Whitney’s U-test were used to compare continuous variables across 2 groups, and chi-square test was used to compare the distributions of categorical variables. A 2-tailed *P*-value cut-off of 0.05 was used as a threshold of statistical significance. All analyses were performed using SPSS v. 27 (IBM, Armonk, NY).

## Results

### Demographic and Clinical Characteristics

We included 67 patients (37 women (55%), 30 men; median age: 37 [IQR: 18–60] years, minimum–maximum range: 2–91 years) who received rituximab either as first-line (*n* = 39) or second-line treatment (*n* = 28) for anti-GBM disease. Mean blood pressure was 143/84 (20/13) mmHg. Patients had a median serum creatinine at initial presentation of 416 μmol/l (IQR: 207–630). In patients who were not dialysis-dependent at presentation, median initial serum creatinine was 212 μmol/l (IQR: 92–295) and peak creatinine was 294 μmol/l (IQR: 120–416). A total of 16 cases reported oliguria or anuria and 32 patients (48%) were dialysis-dependent at initial presentation. Median initial creatinine in initially dialysis-dependent patients was 629 μmol/l (IQR: 519–834) with peak creatinine being 795 μmol/l (IQR: 634–1044). Thirty-four cases reported proteinuria, with 9 reporting nephrotic-range proteinuria. Thirty-four cases reported hematuria, all of them having nephritic syndrome, with 12 of these reporting gross hematuria.

Twenty-four patients had a prodromal infection (8 had upper respiratory infection, 6 had pneumonia, 6 had urinary tract infection, 3 had gastrointestinal infection, 1 had COVID-19, and 1 had streptococcal pharyngitis). Twenty-four patients (36%) had DAH, 3 of whom had isolated DAH. Data on all patients, separated into adult and pediatric patients are presented in Table 1.

**Table 1:** Demographic, clinical, laboratory, histologic parameters and outcomes in all patients and subdivided into adult and pediatric patients

Parameters	All patients (*n* = 67)	Adult patients (*n* = 53)	Pediatric patients (*n* = 14)
Sex (F/M)	37/30	29/24	8/6
Age, yrs (IQR)	37 (18–60)	51 (24–63)	16 (15–17)
Diffuse alveolar hemorrhage, *n* (%)	24 (36)	18 (34)	6 (43)
Initial creatinine (μmol/l)	416 (207–630)	502 (283–707)	169 (66–230)
Creatinine at last follow-up (μmol/l)	123 (83–238)	140 (117–255)	82 (72–98)
Dialysis-dependency at initial presentation, *n* (%)	32 (48)	30 (57)	2 (14)
Anti-GBM antibody levels (IU/ml)	189 (58–258)	190 (47–258)	189 (138–244)
Anti-GBM antibody titer, *n* (%)			
≤ 1/40	3 (30)	3 (33)	0 (0)
> 1/40	7 (70)	6 (67)	1 (100)
Anti-GBM antibody negativity, *n* (%)	42 (100)	31 (100)	11 (100)
Positive ANCA	20 (30)	17 (32)	4 (25)
MPO	15 (75)	12 (71)	3 (75)
PR3	0 (0)	0 (0)	0 (0)
MPO and PR3	1 (6)	1 (6)	0 (0)
Nonspecific or not specified	4 (25)	3 (23)	1 (25)
Crescents			
Total, % (IQR)	80 (57–97)	83 (58–100)	72 (56–88)
Cellular, % (IQR)	78 (45–97)	81 (50–100)	64 (26–91)
Glucocorticoids			
Pulse, *n* (%)	56 (85)	44 (85)	12 (86)
Cumulative dose of methylprednisolone (mg)	1500 (1500–3000)	1500 (1500–3000)	2250 (1500–4000)
Oral, *n* (%)	59 (98)	45 (98)	14 (100)
Plasma exchange	62 (93)	48 (91)	14 (100)
Number of plasma exchange sessions	11 (7–16)	10 (6–16)	12 (10–16)
Cyclophosphamide, *n* (%)	36 (54)	27 (51)	9 (64)
Cumulative dose (mg)	2500 (500–4950)	1225 (500–3000)	6300 (3500–8000)
Rituximab			
Dose			
375 mg/m^2^, *n* (%)	34 (54)	25 (50)	9 (69)
1000 mg, *n* (%)	6 (16)	5 (10)	1 (8)
Other or not specified	23 (30)	20 (40)	3 (23)
Dosing schedule, *n* (%)			
Once weekly	55 (82)	44 (83)	11 (79)
Other or not specified	12 (18)	9 (17)	3 (21)
Number of applications, *n* (IQR)	4 (2–4)	4 (2–4)	4 (2–4)
Adverse events related to rituximab use, *n* (%)	11 (16)	5 (9)	6 (43)
Follow-up length, mo (IQR)	9.5 (4.5–24.7)	6.0 (3.0–24.0)	18.0 (6.8–26.6)
ESKD at last follow-up			
Patients with at least 1-yr follow-up, *n* (%)	11 (36)	10 (91)	1 (9)
All patients, *n* (%)	27 (43)	23 (47)	4 (29)
Death, *n* (%)	6 (9)	5 (9)	1 (7)

ANCA, antineutrophil cytoplasmic antibody; ESKD, end-stage kidney disease; F, female; GBM, glomerular basement membrane; IQR, interquartile range; M, male; MPO, myeloperoxidase; PR3, proteinase 3.

### Diagnosis

All patients had positive anti-GBM antibodies at the initial presentation. In 24 cases, the method to detect anti-GBM antibodies was reported. Enzyme-linked immunosorbent assays (*n* = 16) were leading, whereas other methods such as fluorenzyme assay (*n* = 4) and multiplex bead array assays (*n* = 2), chemiluminescent enzyme immunoassay (*n* = 1) were rarely used. In 44 cases, the specific method was not reported. Antibody status at follow-up was available for 41 patients, all of whom had negative anti-GBM antibodies. Twenty patients were double-positive for anti-GBM antibodies and ANCA, with 15 of them being positive for myeloperoxidase-ANCA alongside anti-GBM antibodies, 3 with positive ANCA without further specification, 1 patient with myeloperoxidase- and proteinase 3–ANCA positivity, and 1 patient with “non-specific ANCA positivity.”

Kidney biopsy was performed in 49 patients. A total of 42 patients (89%) had crescentic glomerulonephritis, with a median proportion of crescents of 80% (57%–97%), which was reported in 41 studies and 78% (45%–97%) cellular crescents, which was reported in 25 studies. Out of 49 cases with performed biopsy, 6 cases reported GBM breaks, 11 reported fibrinoid necrosis, four reported Bowman’s capsule rupture, and 15 reported interstitial inflammation. Six patients had none or minimal interstitial fibrosis and tubular atrophy (≤ 10%), 9 had mild (11%–25%), 4 had moderate (26%–50%), and none had severe (> 50%) interstitial fibrosis and tubular atrophy. All patients showed linear IgG staining along the GBM, 31 had positive C3 staining, 2 had positive IgM staining, whereas 2 each had positive C1q and IgA staining. Fifteen patients had positive staining for light chains, all positive for both kappa and lambda light chains.

### Therapy

Fifty-six patients (85%, with 1 study not reporting on previous glucocorticoid therapy) received pulse methylprednisolone with a median cumulative dose of 1500 mg (IQR: 1500–3000 mg), and 59 out of 60 patients (98%, not reported for 7 patients) received an initial oral glucocorticoid therapy (prednisone equivalent), almost all at a dose of 1 mg/kg body weight. The majority (93%) received PLEX with a median number of 11 (7–16) sessions, whereas 1 patient received immunoadsorption. Thirty-six patients received cyclophosphamide, either before (*n* = 28), concomitant with (*n* = 6), or after rituximab (*n* = 2), with a median cumulative dose of 2500 mg (IQR: 500–4950mg). One patient received avacopan, an oral complement C5aR1 inhibitor approved for the management of ANCA-associated vasculitis.

Rituximab was given after a median of 16 days (7–34) from the day of hospitalization, most commonly as 375 mg/m^2^ (*n* = 34) weekly for 4 total applications or as 1000 mg (*n* = 6) a fortnight apart, with several other, mainly lower-dose regimens, also given. The main indications for first-line rituximab were reported in 31 cases. These were severe disease (*n* = 18) and fertility preservation (*n* = 8),whereas 1 study with 5 patients specifically wanted to test rituximab as first-line in anti-GBM disease. The indications for second-line treatment were reported in all cases and were severe or refractory disease (*n* = 16), relapse (*n* = 8), thrombocytopenia (*n* = 2), and leukopenia (*n* = 2).

Eleven patients (16%) experienced adverse events following rituximab, which was more common in patients with rituximab as the first-line therapy (26% vs. 4%, *P* = 0.017). The most common reported side effect was posterior reversible encephalopathy syndrome, occurring 48 hours after the first dose of rituximab in a patient after discharge, together with *Pneumocystis jirovecii* pneumonia in another, and weeks after the last rituximab infusion in a third patient. Two patients had an allergic reaction during administration, whereas 2 more developed transient leukopenia, the latter a potential carry-over effect of other immunosuppressants. In 1 patient each, esophageal candidiasis with transient thrombocytopenia, mild upper respiratory infection, candida colonization, septic arthritis, progressive leukoencephalopathy occurring 5 weeks after a single dose administration and a prolonged B-cell depletion beyond 9 months were reported. One pediatric patient who had common variable immunodeficiency (CVID) had cytomegalovirus meningoencephalitis after discharge, which occurred several weeks after rituximab. All patients recovered from complications, but the patient with CVID later died of another infection (details are described in the next section).

Comparing first-line and second-line rituximab treatment, patients with first-line treatment had a higher proportion of dialysis-dependence at initial presentation (59% vs. 32%, *P* = 0.03), higher initial serum creatinine (486 vs. 256 μmol/l, *P* = 0.03), and a higher percentage of cellular crescents (93% vs. 35%, *P* = 0.009), but received fewer sessions of PLEX (9 vs. 14, *P* = 0.017) (Table 2).

**Table 2:** Differences in demographic, clinical, laboratory, histologic parameters and outcomes in patients receiving rituximab in the first or second-line

Parameters	First-line rituximab (*n* = 39)	Second-line rituximab (*n* = 28)	*P*-value
Sex (F/M)	23/16	14/14	0.47
Age, yrs (IQR)	38 (18–58)	22 (18–63)	0.88
Diffuse alveolar hemorrhage, *n* (%)	16 (41)	8 (29)	0.30
Initial creatinine, μmol/l (IQR)	486 (276–707)	256 (142–536)	0.03
Creatinine at last follow-up, μmol/l (IQR)	123 (86–242)	123 (83–220)	0.65
Dialysis-dependency at initial presentation, *n* (%)	23 (59)	9 (32)	0.03
Anti-GBM antibody levels, IU/ml (IQR)	189 (46–327)	195 (78–200)	0.89
Anti-GBM antibody titer, *n* (%)			
≤ 1/40	2 (33)	1 (25)	0.67
> 1/40	4 (67)	3 (75)	
Anti-GBM antibody negativity, *n* (%)	21 (100)	21 (100)	>0.99
Positive ANCA, *n* (%)	14 (36)	6 (19)	0.20
MPO, *n* (%)	10 (71)	5 (83)	0.96
PR3, *n* (%)	0 (0)	0 (0)	
MPO and PR3, *n* (%)	1 (7)	0 (0)	
Nonspecific or not specified, *n* (%)	3 (21)	1 (17)	
Crescents			
Total, % (IQR)	82 (58–100)	80 (55–91)	0.63
Cellular, % (IQR)	93 (66–100)	35 (18–59)	0.009
Glucocorticoids			
Pulse, *n* (%)	32 (84)	24 (86)	0.87
Cumulative dose of methylprednisolone, mg (IQR)	1500 (1500–1750)	2250 (1500–3000)	0.10
Oral, *n* (%)	31 (97)	28 (100)	0.34
Plasma exchange, *n* (%)	32 (89)	26 (96)	0.29
Number of plasma exchange sessions, *n* (IQR)	9 (5–15)	14 (10–20)	0.017
Cyclophosphamide, *n* (%)	8 (21)	28 (100)	<0.001
Cumulative dose, mg (IQR)	1000 (500–1450)	3000 (625–6525)	0.18
Rituximab			
Dose			
375 mg/m^2^, *n* (%)	16 (41)	18 (64)	0.18
1000 mg, *n* (%)	3 (8)	3 (11)	
Other or not specified	20 (51)	7 (25)	
Dosing schedule, *n* (%)			
Once weekly	30 (77)	25 (89)	0.19
Other or not specified	9 (23)	3 (11)	
Number of applications, *n* (IQR)	2 (2–4)	4 (2–4)	0.10
Adverse events related to rituximab use, *n* (%)	10 (26)	1 (4)	0.017
Follow-up length, mo (IQR)	7.0 (3.0–24.3)	12.2 (5.1–27.8)	0.37
ESKD at last follow-up			
Patients with at least 1-yr follow-up, *n* (%)	6 (40)	5 (31)	0.62
All patients, *n* (%)	20 (54)	7 (26)	0.03
Death, *n* (%)	6 (15)	0 (0)	0.08

ANCA, antineutrophil cytoplasmic antibody; ESKD, end-stage kidney disease; F, female; GBM, glomerular basement membrane; IQR, interquartile range; M, male; MPO, myeloperoxidase; PR3, proteinase 3.

First-line therapy is defined as patients who did not receive cyclophosphamide previously in this episode of disease, while concomitant use of cyclophosphamide and rituximab was allowed.

Second-line therapy was defined as patients who have received at least 1 dose of cyclophosphamide before rituximab in this episode of disease.

### Outcomes

**Figure 2:**
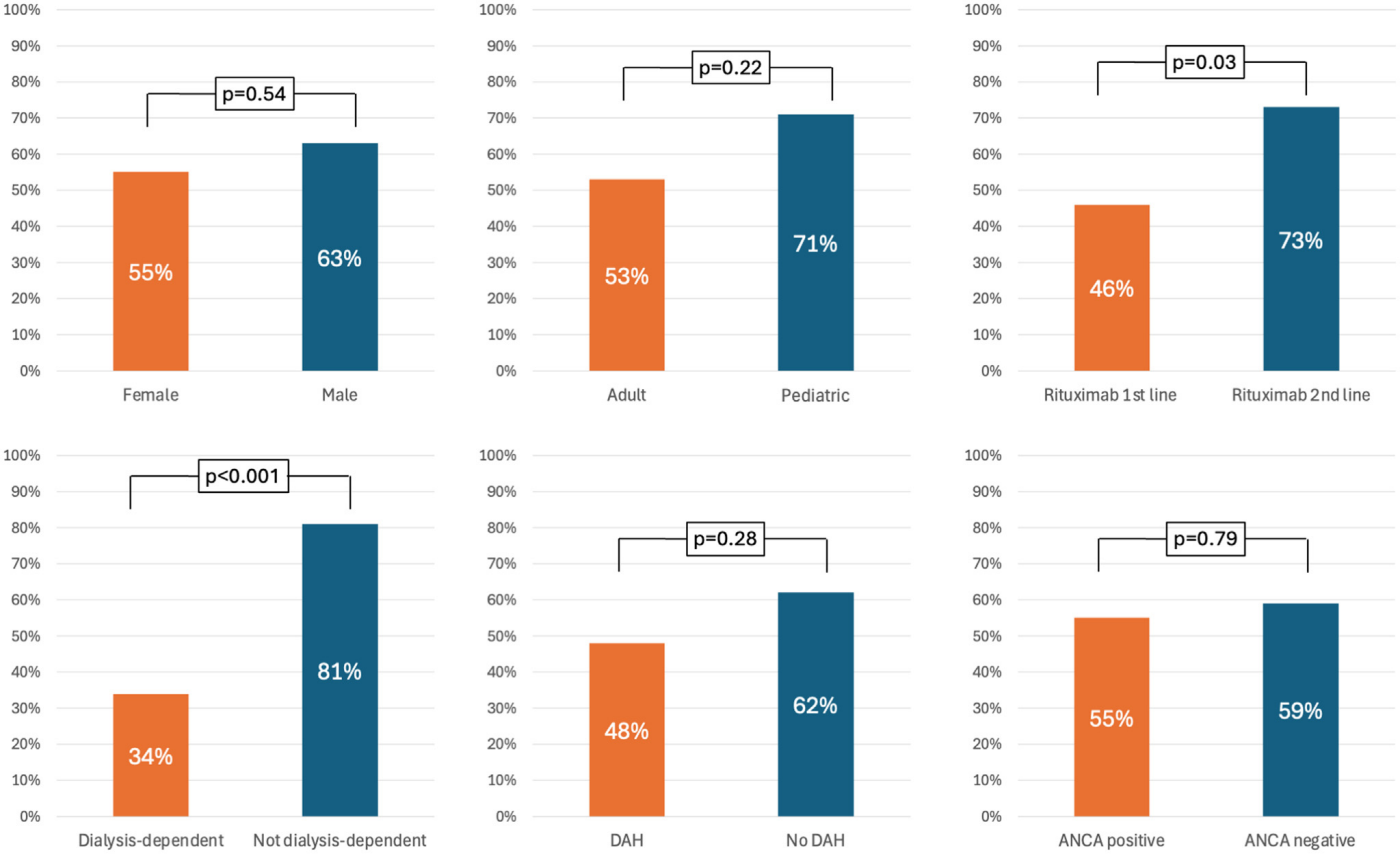
ESKD–free survival at last follow-up in different subgroups. ANCA, antineutrophil cytoplasmic antibodies; DAH, diffuse alveolar hemorrhage; ESKD, End-stage kidney disease.

Follow-up time was available for 64 patients who were followed-up for a total of 87.1 person-years, with a median follow-up time of 9.5 (IQR: 4.5–24.7) months. Of the patients with kidney involvement at baseline (*n* = 63), 27 progressed to end-stage kidney disease, resulting in 57% kidney survival at last follow-up. Serum creatinine decreased to 122 μmol/l (IQR: 79–150) in non–dialysis-dependent patients and to 225 μmol/l (IQR: 97–300) in those who required kidney replacement therapy at baseline. Patients who were dialysis-dependent at initial presentation had worse kidney survival at the last follow-up (34% vs. 81%, *P* < 0.001). There was no significant difference in kidney survival at the last follow-up between ANCA-positive and ANCA-negative patients (55% vs. 59%, *P* = 0.79) and patients with or without DAH (48% vs. 62%, *P* = 0.28). Patients receiving rituximab as the first-line therapy had worse kidney survival than those receiving it as second-line (46% vs. 73%, *P* = 0.03) (Figure 2).

A total of 6 patients (5 adult, one pediatric) died, indicating a patient survival of 91%. All six deaths happened in patients who received first-line rituximab (85% vs. 100%; *P* = 0.08). Three patients died of DAH, of whom 2 also had sepsis, with mortality occurring at an age of 41 and 60 years. Of these, death occurred 2 to 7 weeks after the last dose of rituximab. The 2 other adult patients (aged 52 and 63 years) died 9 and 29 months, respectively after the last dose of rituximab at home because of unknown causes. The pediatric patient, aged 17 years, had CVID and lifelong recurrent respiratory tract infections with multiple complications, including quadriparesis and cognitive changes because of previous meningoencephalitis, which occurred several years before anti-GBM disease was diagnosed. After hospitalization for anti-GBM disease, she had several infections, including cytomegalovirus meningoencephalitis several weeks after rituximab. She died several months after receiving the second and last dose of rituximab, most likely from lower respiratory tract infection. None of the deaths were plausibly related to rituximab.

## Discussion

The 2021 Kidney Disease: Improving Global Outcomes guidelines suggest rituximab as a potential therapeutic option in refractory anti-GBM disease, but mention the paucity of evidence and point out that studies comparing rituximab with cyclophosphamide are needed.^17^ Our review of cases receiving rituximab for anti-GBM disease shows that patients had excellent overall survival with a relatively good kidney survival and safety profile. However, there is a substantial risk of both publication and selection bias. Selection bias is indicated by the relatively young age of this cohort, mainly corresponding to the so-called first peak of this biphasic disease. Patients who were given rituximab as second-line therapy had less severe kidney disease as expressed by the percentage of crescents in the kidney biopsy, proportion of initial dialysis-dependency and initial serum creatinine. Patients receiving second-line rituximab also received more sessions of PLEX. Fertility preservation and lesser risk of developing malignancy also tend to favor directing young persons to this treatment. In addition, when prescribing rituximab as a second-line treatment, younger patients might have been selected because the physicians might otherwise have decided to stop treatment completely.

Besides 6 fatalities, side effects were likely underreported. A total of 11 patients had adverse events, all of which were transient and from which all but 1 patient with CVID fully recovered. The outcome of patients categorized as dialysis-dependent at initial presentation is better in this study than from historic reports. This might be due to a better kidney function at presentation, and we speculate that the indication for dialysis in some of the included cases must have been a relative one. Still, we observed a striking difference in kidney survival between patients who were dialysis-dependent at initial presentation and those who were not, which is in line with previous studies.^17^^,^^18^ Rituximab showed comparable patient and kidney survival to cyclophosphamide in both dialysis-dependent and dialysis-independent patients.^18^, ^19^, ^20^, ^21^

It is interesting to note that, in the 26 patients in whom rituximab was used as second-line therapy (excluding 2 patients with isolated DAH), all of whom failed cyclophosphamide, kidney survival after second-line rituximab was still 44% in dialysis-dependent patients and 88% in non–dialysis-dependent patients, providing promise when the current standard of care management was not successful. However, dissection of the true effects of rituximab from carry-over effects of other used therapies such as cyclophosphamide, glucocorticoids, or PLEX remains difficult.

DAH was much more frequent in patients included in our analysis than in the rituximab arm of the cohort by Chen *et al.* (24 patients in our analysis [36%] vs. 1 (7%) patient) which shows that we have included patients with more severe disease.^16^ However, kidney survival was comparable between patients included in our review and those included in the above-mentioned large study (57% vs. 50%). Moreover, we did not find a difference in survival between patients with and without DAH. The only other study, which involved a substantial number of patients on rituximab was a retrospective study by Marques *et al.* This study included a subset of 11 patients (9 of whom are included in our review) receiving rituximab out of a total of 119 patients, recruited from 11 French centers. The aim of their study was to examine prognostic factors in anti-GBM disease and did not analyze potential benefits of rituximab therapy. Indeed, the authors did not report on any specific safety or other signals.^6^

The Rituximab in ANCA-Associated Vasculitis trial suggested that rituximab might be more efficient than cyclophosphamide for stopping antibody production in ANCA-associated vasculitis, because a higher proportion of patients became ANCA-negative by 6 months (47% vs. 24%, *P* = 0.004).^14^ Previous experimental research identified principal conformational or nonlinear epitopes localized to the globular noncollagenous domain of the α3 chain of type IV and antibodies (predominantly of IgG class) which bind to them, whereas pathogenicity has been demonstrated by passive transfer experiments and indirectly in clinical studies in which levels were associated with kidney outcomes.^22^, ^23^, ^24^, ^25^, ^26^, ^27^ Given the demonstrated pathogenicity of anti-GBM antibodies, the ultimate goal of treatment is rapid reduction of circulating autoantibodies. The results of the recently reported phase 2a trial GOOD-IdeS-01 showed that imlifidase, an agent based on the Ig-degrading enzyme of *Streptococcus pyogenes* (IdeS) which cleaves all IgG within several hours, is associated with better outcomes than historic cohorts. All of this might, from a pathophysiologic perspective, make rituximab a logical candidate for the treatment of anti-GBM disease.^28^^,^^29^ Recent evidence by Chen *et al.* might support this, because patients treated with rituximab + PLEX had a faster antibody level decline than those treated with cyclophosphamide + PLEX (75.3% ± 17.6% vs. 56.3% ± 18.3%, *P* = 0.018). Moreover, patients progressing to end-stage kidney disease also had a faster decline than those who did not.^16^

Although there are no studies evaluating the use of rituximab in double-positive patients, a recent review examining the characteristics, treatment, and prognosis of double-positive patients reported its use in induction therapy in several patients and posed it as a potential therapeutic option in these patients.^30^ In our review, there were 20 patients who were ANCA-positive. There were no differences in prognosis of double-positive patients compared with solely anti-GBM positive patients. Given the good results of rituximab in ANCA-associated vasculitis, especially in proteinase 3–ANCA positive patients, it might be postulated that it would be efficacious in these patients too.

Finally, our study included 14 pediatric patients, which makes it one of the largest cohorts of pediatric anti-GBM disease reported so far. Children in our group had comparable outcomes with those previously reported.^5^^,^^31^^,^^32^ Adverse events were more common in children, but all were transient and the only child who died had CVID and died several months after rituximab.

Our review has several limitations, most notably the relatively small number of cases; however, it contains by far the largest number of anti-GBM disease patients treated with rituximab. The review presents a collection of cases and thus, represents a very heterogenous population; bias toward reporting positive outcomes of single case reports and case series cannot be ruled out. A major limitation is the lack of establishing and describing specific time courses on achieving certain endpoints or chronology of change in key variables. Furthermore, many included studies did not contain a number of relevant variables, and the vast majority did not report time-to-event, so we could not calculate precise survival curves, only the proportions of patients with an event at the end of follow-up. We have tried to overcome this by reporting end-stage kidney disease for all patients and patients with at least 1-year follow-up, and by reporting multiple values, that is, serum creatinine (at the initial encounter, peak, and at the time of last follow-up). Other points to consider are completeness of data and reporting bias. Although we have strived to extract all relevant information, some cases might not have presented all information on the patients and in these cases we coded these as missing values and could not achieve completeness for these variables. This is especially the case with bias in not reporting negative results, where cases had a much higher tendency to report on some key findings (e.g., DAH or no DAH) and positive findings (e.g., presence of proteinuria or hematuria) than lack of such findings (e.g., no proteinuria).

Smoking has been previously recognized as a potential risk factor for anti-GBM disease and especially for pulmonary involvement. This might be explained by the more frequent underlying pulmonary injury in smokers, which could lead to uncovering previously cryptic antigens which would then induce autoantibody formation.^33^, ^34^, ^35^ However, case reports did not generally report on smoking, and we could not include it in analysis. Furthermore, we were not able to include information on hypogammaglobulinemia and neutropenia, which are important points to consider in the link between rituximab and infectious complications and might lead to hesitancy in giving rituximab.^36^, ^37^, ^38^ The Rituximab in ANCA-Associated Vasculitis trial, which included patients with ANCA-associated vasculitis, showed no difference in the risk of infection between rituximab and cyclophosphamide; and a very recent analysis of long-term surveillance of rituximab, which included 392 patients with ANCA-associated vasculitis (247 receiving rituximab) found that patients receiving rituximab have more than double the risk of serious infections compared with controls receiving other immunosuppressive therapies.^14^^,^^39^Although we have explored complications of rituximab in this study, including infectious ones, only few case reports provided data on hypogammaglobulinemia or neutropenia. Moreover, we report only on patients treated with rituximab and we did not have a control cohort treated with conventional therapy. Thus, our results are mainly descriptive from this perspective and any comparisons with other treatments could be done qualitatively and using historical findings from other studies. However, we have here presented the largest series of patients with anti-GBM treated with rituximab and provide evidence on its efficacy and safety. Although it is difficult to conduct randomized controlled trials in anti-GBM disease, with GOOD-IdeS-02 being 1 of the only 2 exceptions, large prospective and retrospective studies including head-to-head comparisons with other treatment arms are much warranted.

To conclude, our study shows that, given the apparently similar efficacy, rituximab might be considered as second-line therapy in anti-GBM disease when further use of cyclophosphamide is contraindicated and potentially as first-line therapy in patients with mild disease or otherwise not suitable for initiation of cyclophosphamide.

## Disclosure

IB has received consultancy fees from Amgen, Q32 Bio, Aurinia, GSK, Hansa, and Alentis; research funding from Novartis; and educational funding from Vifor. IB is the director of BiPath and member of Novartis Advisory board for glomerular disease.AB has received consultancy fees from Alexion, AstraZeneca, Boehringer Ingelheim, and CSL-Vifor; and speakers’ honoraria from Amgen, AstraZeneca, Bayer, Boehringer Ingelheim, GSK, CSL Vifor, and Otsuka. AB is an Editorial Board member of CKJ.VI has received support from the European Renal Association (ERA) Long-term Fellowship.RK is funded by the Medical and Clinician Scientist Program (MCSP) of the Medical Faculty of LMU Munich.AK has received consulting fees or honoraria for 10.13039/100002429Amgen, 10.13039/100004325AstraZeneca, 10.13039/100008349Boehringer Ingelheim, CSL Vifor, Delta4, GlaxoSmithKline, Novartis, Otsuka, Roche, Sobi, and Walden Biosciences. AK is an Editorial Board member of Glom Dis and Nephrol Dial Transplant.SPM has received research funding from AstraZeneca, Senya Therapeutics, and Therini Bio; and consulting fees or honoraria from Alexion, AstraZeneca, CSL Vifor, GlaxoSmithKline, and Travere Therapeutics. SPM is the UK coordinating investigator for the GOOD-IDES-02 trial sponsored by Hansa Pharmaceuticals. MS has received grants from Swedish Kidney Foundation, Skane University, and Swedish Government (ALF). MS has received consulting fees from Hansa Biopharma and Vifor Pharma and payment honoraria from Astellas. MS has participated on a Novartis Advisory Board. All the other authors declared no competing interests.

## Supplementary Material

Supplementary File (PDF) List of Included Studies.

## Footnotes

Supplementary File (PDF)
List of Included Studies.
