# Supplementary material for: Efficacy and Safety of Rituximab in Antiglomerular Basement Membrane Disease
Source: Kidney Int Rep. 2024 Dec 31;10(3):743–52. doi: 10.1016/j.ekir.2024.12.026 (PMC11993202; doi:10.1016/j.ekir.2024.12.026)
Supplement: Supplementary File (PDF) — List of Included Studies. [file mmc1.pdf]

List of included studies

A Studies reporting patient-level data

1. Tanaka R, Toishi T, Masaki R, Aihara H, Sakamoto S, Ikeda M, Inoue T, Kawaji A, Matsunami M, Fukuda J, Ohara M, Kuji H, Ichikawa D, Suzuki T. Effective management of necrotizing crescentic glomerulonephritis using an aggressive combination therapy including avacopan in a patient double-seropositive for anti-GBM antibodies and ANCA: a case report. CEN Case Rep. 2024 Sep 10. doi: 10.1007/s13730-024-00929-4. Epub ahead of print. PMID: 39254789.
2. Arnaert S, Schepens N, Deleu L, Malfait T. Case report: renal recovery in Goodpasture's syndrome treated with rituximab. J Nephrol. 2024 Jun;37(5):1367-1370. doi: 10.1007/s40620-024-01892-0. Epub 2024 Mar 1. PMID: 38427310.
3. Kunaprayoon L, Scheffel ETC, Abdel-Rahman EM. Management of Double-Seropositive Anti-Glomerular Basement Membrane and Anti-Neutrophil Cytoplasmic Antibodies with 100% Crescentic Glomerulonephritis and Nephrotic Range Proteinuria in a Young Female. Biomedicines. 2024 Apr 19;12(4):906. doi: 10.3390/biomedicines12040906. PMID: 38672260; PMCID: PMC11048036.
4. Kanaoka K, Ihara S, Nakatani T, Minami S. Rituximab for the Treatment of Anti-glomerular Basement Membrane Disease with Isolated Diffuse Alveolar Hemorrhage. Intern Med. 2023 Jul 1;62(13):1971-1975. doi: 10.2169/internalmedicine.0166-22. Epub 2022 Oct 19. PMID: 36261370; PMCID: PMC10372275.
5. Yang XF, Jia XY, Yu XJ, Cui Z, Zhao MH. Rituximab for the treatment of refractory anti-glomerular basement membrane disease. Ren Fail. 2022 Dec;44(1):1123-1129. doi: 10.1080/0886022X.2022.2097405. PMID: 35820833; PMCID: PMC9291707.
6. Nalcacioglu H, Tekcan D, Meydan BC, Onal HG, Aydog O. Macroscopic hematuria, facing an uncommon disease: Answers. Pediatr Nephrol. 2022 Feb;37(2):339-343. doi: 10.1007/s00467-021-05285-4. Epub 2021 Oct 19. PMID: 34668062.
7. Al-Chalabi S, Wu HHL, Chinnadurai R, Ponnusamy A. Etanercept-Induced Anti-Glomerular Basement Membrane Disease. Case Rep Nephrol Dial. 2021 Sep 21;11(3):292-300. doi: 10.1159/000518984. PMID: 34722648; PMCID: PMC8543357.
8. Winkler A, Zitt E, Sprenger-Mähr H, Soleiman A, Cejna M, Lhotta K. SARS-CoV-2 infection and recurrence of anti-glomerular basement disease: a case report. BMC Nephrol. 2021 Feb 27;22(1):75. doi: 10.1186/s12882-021-02275-4. PMID: 33639869; PMCID: PMC7914035.
9. Goda S, Gando S, Berg BW. Veno-venous extracorporeal membrane oxygenation (VV-ECMO) for life-threatening isolated pulmonary anti-GBM disease. Respir Med Case Rep. 2022 Jun 1;38:101680. doi: 10.1016/j.rmcr.2022.101680. PMID: 35677578; PMCID: PMC9168115.
10. Helander L, Hanna M, Annen K. Pediatric double positive anti-glomerular basement membrane antibody and anti-neutrophil cytoplasmic antibody glomerulonephritis-A case report with review of literature. J Clin Apher. 2021 Jun;36(3):505-510. doi: 10.1002/jca.21886. Epub 2021 Feb 25. PMID: 33629780.

11. Povey J, Rutherford E, Levy J, Muniraju T. Relapse of treated anti-GBM disease following hair dye use. BMJ Case Rep. 2021 Apr 1;14(4):e240543. doi: 10.1136/bcr-2020-240543. PMID: 33795274; PMCID: PMC8023745.
12. Chittka D, Lennartz L, Jung B, Banas B, Bergler T. Erfolgreiche Rituximabtherapie des Rezidivs einer Glomerulonephritis assoziiert mit Antikörpern gegen die glomeruläre Basalmembran [Successful rituximab treatment of recurrent glomerulonephritis associated with antibodies against the glomerular basement membrane]. Internist (Berl). 2020 Apr;61(4):416-423. German. doi: 10.1007/s00108-020-00773-5. PMID: 32179970.
13. Uematsu-Uchida M, Ohira T, Tomita S, Satonaka H, Tojo A, Ishimitsu T. Rituximab in treatment of anti-GBM antibody glomerulonephritis: A case report and literature review. Medicine (Baltimore). 2019 Nov;98(44):e17801. doi: 10.1097/MD.0000000000017801. PMID: 31689860; PMCID: PMC6946414.
14. Timmermans SAMEG, van Dam MJCM, Vink E, Horuz FAPT, van Paassen P, Rosias PPR. Rituximab for the Treatment of Pediatric Double-Positive Small-Vessel Vasculitis. Kidney Int Rep. 2019 Nov 26;5(2):235-238. doi: 10.1016/j.ekir.2019.11.009. PMID: 32043039; PMCID: PMC7000840.
15. Mannemuddhu SS, Clapp W, Modica R, Elder ME, Upadhyay K. End-stage renal disease secondary to anti-glomerular basement membrane disease in a child with common variable immunodeficiency. Clin Nephrol Case Stud. 2019 Feb 1;7:1-6. doi: 10.5414/CNCS109510. PMID: 30838168; PMCID: PMC6374989.
16. Jain R, Dgheim H, Bomback AS. Rituximab for Anti-Glomerular Basement Membrane Disease. Kidney Int Rep. 2018 Dec 17;4(4):614-618. doi: 10.1016/j.ekir.2018.12.002. PMID: 30993238; PMCID: PMC6451084.
17. Heitz M, Carron PL, Clavarino G, Jouve T, Pinel N, Guebre-Egziabher F, Rostaing L. Use of rituximab as an induction therapy in anti-glomerular basement-membrane disease. BMC Nephrol. 2018 Sep 20;19(1):241. doi: 10.1186/s12882-018-1038-7. PMID: 30236081; PMCID: PMC6149204.
18. Verdesca SVS, Villani C, Rossini M, Manno C, Gesualdo L, Montinaro V. [Posterior Reversible Encephalopathy Syndrome (PRES) induced by Rituximab in two patients with vasculitis, and treated by hemodialysis]. G Ital Nefrol. 2018 Jul;35(4):2018-vol4. Italian. PMID: 30035447.
19. Lemahieu W, Ombelet S, Lerut E, Jamar S, Sprangers B. Reversal of Dialysis-Dependent Anti-Glomerular Basement Membrane Disease Using Plasma Exchange, Glucocorticosteroids, and Rituximab. Kidney Int Rep. 2018 May 8;3(5):1229-1232. doi: 10.1016/j.ekir.2018.04.015. PMID: 30197991; PMCID: PMC6127412.
20. Huang J, Wu L, Huang X, Xie Y, Yu J, Yang J, Fang H, Zhang L. Successful Treatment of Dual-Positive Anti-Myeloperoxidase and Anti-Glomerular Basement Membrane Antibody Vasculitis with Pulmonary-Renal Syndrome. Case Rep Nephrol Dial. 2016 Jan 8;6(1):1-7. doi: 10.1159/000443163. PMID: 26889474; PMCID: PMC4748756.
21. Touzot M, Poisson J, Faguer S, Ribes D, Cohen P, Geffray L, Anguel N, François H, Karras A, Cacoub P, Durrbach A, Saadoun D. Rituximab in anti-GBM disease: A retrospective study of 8 patients. J Autoimmun. 2015 Jun;60:74-9. doi: 10.1016/j.jaut.2015.04.003. Epub 2015 May 4. PMID: 25953709.

22. Narayanan M, Casimiro I, Pichler R. A unique way to treat Goodpasture's disease. BMJ Case Rep. 2014 Nov 24;2014:bcr2014206220. doi: 10.1136/bcr-2014-206220. PMID: 25422333; PMCID: PMC4244353.
23. Syeda UA, Singer NG, Magrey M. Anti-glomerular basement membrane antibody disease treated with rituximab: A case-based review. Semin Arthritis Rheum. 2013 Jun;42(6):567-72. doi: 10.1016/j.semarthrit.2012.10.007. Epub 2013 Jan 24. PMID: 23352254.
24. Shah Y, Mohiuddin A, Sluman C, Daryanani I, Ledson T, Banerjee A, Crowe A, McClelland P. Rituximab in anti-glomerular basement membrane disease. QJM. 2012 Feb;105(2):195-7. doi: 10.1093/qjmed/hcr001. Epub 2011 Jan 21. PMID: 21258056.
25. Mutsaers P, Selten H, van Dam B. Additional antibody suppression from rituximab added to conventional therapy in severe, refractory anti-GBM nephritis. NDT Plus. 2010 Aug;3(4):421-2. doi: 10.1093/ndtplus/sfq085. Epub 2010 May 13. PMID: 25949452; PMCID: PMC4421516.
26. Schless B, Yildirim S, Beha D, Keller F, Czock D. Rituximab in two cases of Goodpasture's syndrome. NDT Plus. 2009 Jun;2(3):225-7. doi: 10.1093/ndtplus/sfp020. Epub 2009 Feb 18. PMID: 25983996; PMCID: PMC4421183.
27. Arzoo K, Sadeghi S, Liebman HA. Treatment of refractory antibody mediated autoimmune disorders with an anti-CD20 monoclonal antibody (rituximab). Ann Rheum Dis. 2002 Oct;61(10):922-4. doi: 10.1136/ard.61.10.922. PMID: 12228164; PMCID: PMC1753910.

B Studies in which patient-level data were obtained from authors

1. Klaus R, Kanzelmeyer N, Haffner D, Lange-Sperandio B. Outcome of rituximab treatment in children with non-dialysis-dependent anti-GBM disease. Pediatr Nephrol. 2024 Sep 25. doi: 10.1007/s00467-024-06512-4. Epub ahead of print. PMID: 39320552.
2. Kumar A, Gupta S, Jarial KDS, Sangha S, Chauhan A, Sharma V, Sandal R, Sharma D. Clinical Profile and Renal Survival of Anti-Glomerular Basement Membrane Disease Patients: A Retrospective Case Series from Northern India. Glomerular Dis. 2023 Oct 17;3(1):241-247. doi: 10.1159/000534498. PMID: 38021463; PMCID: PMC10645438.
3. Jaryal A, Vikrant S. Anti-glomerular basement membrane disease: Treatment outcome of cyclophosphamide vs. rituximab induction therapy regimen. Clin Nephrol. 2022 Dec;98(6):280-287. doi: 10.5414/CN110851. PMID: 36282172.
4. Marques C, Carvelli J, Biard L, Faguer S, Provôt F, Matignon M, Boffa JJ, Plaisier E, Hertig A, Touzot M, Moranne O, Belenfant X, Annane D, Quéméneur T, Cadranel J, Izzedine H, Bréchot N, Cacoub P, Piedrafita A, Jourde-Chiche N, Saadoun D. Prognostic Factors in Anti-glomerular Basement Membrane Disease: A Multicenter Study of 119 Patients. Front Immunol. 2019 Jul 18;10:1665. doi: 10.3389/fimmu.2019.01665. PMID: 31396214; PMCID: PMC6662558.
